# Taking account of others’ goals in social information use: Developmental changes in 3- to 7-year-old children

**DOI:** 10.1016/j.jecp.2021.105325

**Published:** 2022-03

**Authors:** Kirsten H. Blakey, Mark Atkinson, Eva Rafetseder, Elizabeth Renner, Christine A. Caldwell

**Affiliations:** Department of Psychology, Faculty of Natural Sciences, University of Stirling, Stirling, FK9 4LA, UK

**Keywords:** Information use, Copying, Goals, Cognitive development, Social learning, Comparative psychology

## Abstract

The ability to take mental states such as goals into account when interpreting others’ behavior has been proposed to be what sets human use of social information apart from that of other animals. If so, children’s social information use would be expected to change as their understanding of others’ mental states develops. We explored age-related changes in 3- to 7-year-old children’s ability to strategically use social information by taking into account another’s goal when it was, or was not, aligned with their own. Children observed as a puppet demonstrator selected a capsule, peeked inside, and chose to accept or reject it, following which children made their own selection. Children were able to account for others’ conflicting motivations from around 4 years of age and reliably inferred the outcome of others’ behavior from 6 years. However, using social information based on such inferences appeared to be challenging regardless of whether the demonstrator’s goal was, or was not. aligned to that of the participant. We found that social information use improved with age; however, this improvement was restricted to cases in which the appropriate response was to avoid copying the demonstrator’s selection. In contrast to previous research, appropriate copying responses remained at chance. Possible explanations for this unexpected pattern of results are discussed. The cognitive challenge associated with the ability to account for others’ goals could offer humans a significant advantage over that of other animals in their ability to use social information.

## Highlights

• Social information use improved with age but only for exploration of alternatives.
• Children were able to account for others’ conflicting motivations from 4 years.
• Alignment of the demonstrator’s and children’s goals did not influence performance.
• From 6 years children could infer the outcome of others’ behaviour.
• Rates of copying were low at all ages, even when it was the appropriate response.

## Introduction

It is widely recognized that, relative to other species, humans are particularly adept in their use of social information (Boyd and Richerson, 1996, Tomasello, 1999, Whiten, 2011). The ability to interpret others’ behavior with reference to underlying mental states such as goals, knowledge, and beliefs is thought to be one way in which the use of social information may differ in humans compared with other animals (Baldwin and Moses, 1996, Dunstone and Caldwell, 2018, Heyes, 2016b, Wood et al., 2013b). That is, these particular ways of using social information are proposed to rely on advanced cognitive capacities that are not available to very young children or nonhuman animals. If this is true, then we should expect to see children’s use of social information change with development, in line with their developing understanding of others’ mental states. In the current study, we investigated how children’s use of social information is influenced by their ability to make inferences from a demonstrator’s actions using information about the demonstrator’s goal. In particular we were interested in whether—and how—this changes with age. If using social information under such circumstances turns out to be challenging for children (e.g., if it appears beyond the capabilities of very young children), this may help to elucidate contexts in which humans are likely to have an advantage over other species in their ability to use social information.

There is strong evidence of selective social information use in both very young children and many nonhuman animal species (e.g., Haun et al., 2013, Horner et al., 2010, Kendal et al., 2015, Laland, 2004, Ottoni et al., 2005, Over et al., 2013, Price et al., 2017, Wood et al., 2016, Wood et al., 2012). However, there appear to be key differences in human adults’ use of social information (relative to other animals) that pose a potentially significant advantage for cultural transmission (Morgan, Rendell, Ehn, Hoppitt, & Laland, 2012). These differences manifest in human adults’ capacity to seek out, attend to, and use social information across a vast array of contexts. This raises the question of whether the ways in which humans use social information are more cognitively complex than those used by animals.

Recognizing the potential value of social information is thought to be cognitively challenging and likely to emerge later during childhood (Dunstone and Caldwell, 2018, Heyes, 2016a). Although individuals may appear as though they are making strategic decisions regarding social information use, we propose that both younger children and animals may be more likely to rely on less cognitively demanding processes. For instance, heuristic biases may produce behavior that is superficially similar to that produced by reasoned understanding of states such as knowledge and desire; however, they do not afford the equivalent degree of flexibility. Indeed, biases such as “copy successful individuals” are appropriate only when the goals of both parties are aligned. If goals are not aligned, then copying another’s behavior or selection is unlikely to be successful for a learner even if it has been successful for another.

Understanding whether available social information is valuable and relevant for achieving a specific goal almost certainly requires some degree of representation of others’ goals as well as the capacity to interpret the outcomes of others’ behavior in relation to those goals. Below we discuss the developmental trajectory of sociocognitive and metacognitive abilities thought to be necessary to engage in such social information use.

The ability to recognize and represent others’ goals relative to one’s own goals has primarily been investigated with regard to understanding others’ desires. From as early as 2 years of age, children are reported to be able to recognize and act according to others’ desires or preferences on the basis of their actions (e.g., Fawcett and Markson, 2010, Repacholi and Gopnik, 1997). However, such early development of this capacity has not been replicated consistently in the literature. For example, some studies have reported finding that understanding of others’ desires develops closer to 3 years of age (Rakoczy et al., 2007, Ruffman et al., 2018), as was previously suggested by Wellman and Woolley (1990). Furthermore, there is evidence to suggest that children struggle to deal with others’ desires when they conflict with their own until about 5 years of age (Atance et al., 2010, Moore et al., 1995, Moses et al., 2000, Rostad and Pexman, 2014). These studies examined desire understanding in relation to emotional responses, so it is important to consider whether the developmental trajectory is the same for other categories of desires or goals (i.e., abstract or task-specific goals).

One way of examining children’s understanding of task-specific goals is to investigate differences in understanding of others’ diverse desires in cooperative contexts compared with competitive contexts. Cooperation appears to closely align with children’s ability to understand others’ mental states (Priewasser, Roessler, & Perner, 2013). However, children under 5 years of age struggle to report another player’s desire in a competitive game, instead reporting their own desire as that of the other player (Moore et al., 1995). Thus, recognizing the potential for incompatible desires appears to remain cognitively challenging for longer during development in competitive contexts. Direct comparison of cooperative and competitive contexts found that 4-year-olds still have difficulty in understanding others’ diverse desires, and although cooperative contexts aid desire reasoning, competitive contexts appear to pose greater challenges (Jin, Li, He, & Shen, 2017). Competitive contexts may be particularly challenging due to the necessity to reason about another’s conflicting desire and how that can be used to benefit oneself. Cooperative contexts might not require the same level of reasoning and indeed might not require explicit reasoning about goals at all.

Only in some circumstances is an understanding of others’ goals necessary for achieving one’s own goal. For instance, social learning situations in which the reward value of others’ behavior is transparent do not necessitate such understanding. By contrast, understanding whether another’s goal is aligned with one’s own is essential in situations where the reward value is opaque because there are only others’ reactions and choices to go on. However, an ability to understand others’ goals can help to interpret these cues. Although understanding others’ goals is not sufficient for interpreting an opaque outcome, it is necessary for appreciating the link between experience and knowledge. That is, learners will be required to infer that a demonstrator has privileged access to the information that allows them to make an informed choice and thus makes the demonstrator a valuable source of information. This is particularly relevant for the experimental procedure used in the current study.

The ability to attribute epistemic states to others based on their reaction to an opaque outcome likely relies on a sound representation of the others’ goal (i.e., their desired outcome) and an understanding of whether the behavior was knowledge based or not (i.e., whether they made an informed choice). Therefore, younger children may struggle to correctly identify the outcome of others’ behavior despite being able to appropriately represent others’ goals due to an inability to recognize the causal link between information access and privileged knowledge before around 5 years of age (Kloo and Rohwer, 2012, O’Neill et al., 1992, Pillow, 1989, Robinson et al., 2008, Ruffman and Olson, 1989, Sodian et al., 2006). Prior to this, if required to identify others’ epistemic states, they likely rely on less cognitively demanding processes such as a previously formed associative rule or bias. Use of such rules or biases may give the appearance of understanding that someone is knowledgeable due to the person’s access to information when in reality information access has not been considered and instead they have relied on a more salient, less cognitively demanding cue (e.g., others’ success).

Even if children have developed the ability to understand and represent others’ goals and use this understanding to interpret the outcome of their behavior, they might not yet have the capacity to recognize the potential value of such information to themselves for solving a specific problem. To appropriately apply this understanding to their social information use, children need to determine whether another’s goal is aligned with their own. Correctly interpreting the outcome of another’s behavior, but not appropriately applying that understanding to inform information use, may indicate that learners have not recognized its value to themselves. In such cases, learners may be more likely to base decisions on salient cues or heuristic biases such as “copy successful others,” and these decisions may appear as reasoned when their goals align with others (or when copying leads to a successful outcome) but will result in lower success when goals are not aligned.

The aim of the current study was to investigate age-related changes in children’s ability to strategically use social information by taking into account others’ goals relative to their own. We were particularly interested in whether children’s abilities to interpret and use social information were influenced by the degree to which others’ goals aligned with their own. Therefore, we aimed to assess whether it is more cognitively challenging to learn from (or use information provided by) someone who is trying to do something different. Using a social learning scenario, we compared the social information use of children assigned to two different conditions; in one condition the goals of the demonstrator and the participant were aligned (Same Goals condition), whereas in the other condition their goals were not aligned (Different Goals condition). Task goals were related to retrieving a target capsule from one of two locations, each containing a different type of capsule; the target capsule was dependent on the individual’s goal. Participants observed a demonstrator select and peek inside a capsule and then accept it (successful outcome) or reject it (unsuccessful outcome). Both outcomes were potentially equally informative to individuals who had represented others’ goals relative to their own and recognized the value of the information. After observing the demonstrator’s reaction, participants needed to make a binary choice between *copying* the demonstrator’s selection by selecting a capsule from the same location and *shifting* away from the demonstrator’s selection by selecting a capsule from the alternative location. Thus, correct responses could not simply be explained by inclinations to copy; instead, they required a selective response based on the inferences made.

Therefore, in contrast to many previous studies (e.g., Evans et al., 2018, Flynn and Whiten, 2013, Haun et al., 2013, McGuigan, 2013, Renner et al., 2020, van Leeuwen et al., 2018, Want and Harris, 2001, Wood et al., 2013a), success in the current study was dependent on children’s appropriate use of two possible responses: copying and shifting. The appropriate response was dependent on the demonstrator’s goal and the outcome of the demonstration. If the demonstrator had the same goal as the participant, the appropriate response was to copy successful selections and shift following unsuccessful selections. If their goals were different, the appropriate response was to shift after successful selections and copy unsuccessful selections. To adopt an appropriate response, participants needed to represent others’ goals relative to their own, interpret the outcome of the demonstration, and recognize the relevance of this information for achieving their own goal.

We predicted that goal understanding, interpreting the outcome of the demonstration, and appropriate information use would improve with age owing to children’s developing understanding of diverse desires and conflicting motivations. We also expected that children’s performance would be greater in the Same Goals condition compared with the Different Goals condition. The expected difference between the conditions could indicate that children find it more challenging to use social information from another whose goal does not align with their own compared with another whose goal matches their own.

## Method

### Participants

The final participant sample comprised 190 children aged 3 to 7 years (97 girls; *M*_age_ = 65.7 months, *SD* = 16.6). Participants were unsystematically allocated to one of two experimental conditions (Same Goals condition: *n* = 97; Different Goals condition: *n* = 93) subject to the constraint that the number of children in each age group was balanced across conditions. In the final sample, there were 18 to 20 participants per age group (classified according to age in whole years) in each condition. The nationality of the sample, as identified by parents/legal guardians, was predominantly British. Participants were recruited from nurseries, primary schools, and public attractions in Scotland. An additional 15 children were tested but later excluded from analyses due to researcher error (*n* = 3), missing data (*n* = 8), task interference (*n* = 2), or confidential disclosures by the child’s guardian that cast doubt on whether that child’s performance could be considered as representative of the intended recruitment population (*n* = 2). This study was granted ethical approval by the University of Stirling General University Ethics Panel, and informed written consent was provided by all participants’ parents/legal guardians prior to data collection.

### Materials

**Fig. 1:**
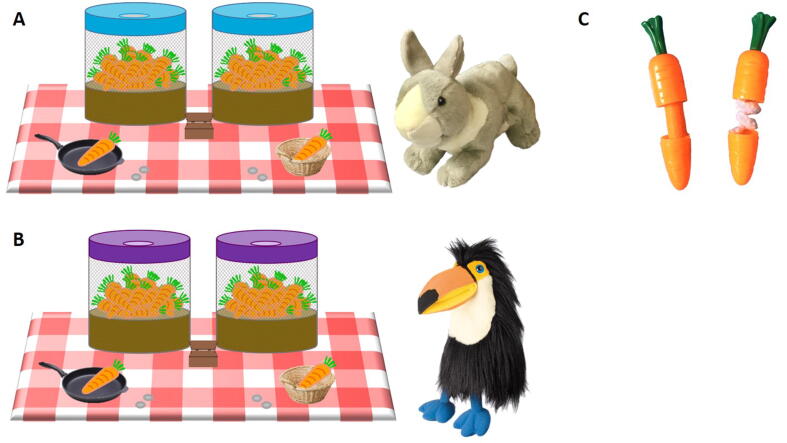
Illustration of the experimental setup. (A) Example setup during a trial in the Same Goals condition with Rabbit as demonstrator. (B) Example setup during a trial in the Different Goals condition with Bird as demonstrator. (C) Example of the carrot capsules opened to reveal the two different content types; the capsules inside one bucket contained orange felt, and the capsules inside the other bucket contained a pink knitted “worm.”

Task apparatus is presented in Fig. 1. Two hand puppets, “Rabbit” (A. S. Puppets) and “Bird” (The Puppet Company Ltd, Hitchin, UK), served as demonstrators. Two round mesh buckets (342 × 235 mm) each contained 23 carrot-shaped plastic capsules (142 × 31 mm) (Fig. 1A and 1B). Each carrot-shaped capsule could be opened to reveal hidden contents (Fig. 1C). The capsules inside one of the buckets contained a roll of orange felt, and the capsules inside the other bucket contained a pink knitted “worm.” Each bucket was fitted with an elasticated fabric lid with an elasticated hole in the center (Fig. 1A and 1B). Within a trial both buckets were fitted with lids of the same color (e.g., both red), and between trials the color of the lids was different (red, purple, and blue). Six gray plastic tokens (23 mm diameter) and a wooden box (580 × 580 × 350 mm) were employed as a “pay to play” system for the demonstrators and participants. The demonstrators stored target capsules in a wicker basket, and participants stored target capsules in a plastic frying pan.

### Design

Participants took part individually in a single session for which they received a sticker. Two experimenters were present; Experimenter 1 (E1) provided instructions and asked all questions (see online supplementary material for a full verbal script), and Experimenter 2 (E2) controlled the demonstrations using their spare hand to assist the puppets’ actions and live-coded all responses. In some sessions a familiar adult was present but was asked not to interact with the participant.

The task comprised a series of three trials completed in one of two conditions. In the Same Goals condition the participant and demonstrator (Rabbit puppet) shared the same task goal; both searched for capsules containing orange felt. By contrast, in the Different Goals condition the participant and demonstrator (Bird puppet) had different task goals; the participant searched for capsules containing orange felt, whereas the demonstrator searched for capsules containing a worm. Across both conditions the format of all trials was the same. Each trial included a social demonstration in which the demonstrator selected a capsule from one of the two buckets and “peeked” inside without letting the participant see the contents. Demonstrations were either successful (demonstrator selected and accepted a target capsule) or unsuccessful (demonstrator selected and rejected a nontarget capsule); the target capsule was dependent on the condition. Each participant experienced a combination of successful and unsuccessful demonstrations. The combination and order of the demonstrations were randomly assigned, subject to the constraint that each participant observed at least one successful and one unsuccessful demonstration across the three trials. The demonstrator accepted the capsule by placing it in the basket or rejected it by giving it to E1. Rejected capsules were given to E1 rather than being returned to the buckets so as to remove the risk of participants returning capsules to the wrong bucket, causing potential confusion in following trials, and to avoid a noticeable difference in the quantity of capsules in each bucket.

After each trial the buckets were removed from the table and the lids were replaced with lids of a different color, the buckets were then returned to the table for the next trial. The sides on which the buckets were presented (distinguished by the contents of the capsules) were randomly allocated in each trial. Following the third trial participants were asked to provide explicit verbal reasoning for their selections: “How were you deciding which buckets to choose?”

### Procedure

Participants were told that they were going to play a game with Rabbit (Same Goals condition) or Bird (Different Goals condition). Between conditions the procedure varied only to accommodate the demonstrators’ alternative goals. E1 presented the two buckets of carrot-shaped capsules and told participants that in one of the buckets the carrots were orange inside and in the other bucket the carrots had a worm inside; examples of each capsule type were presented alongside the explanation.

Each trial began with participants being informed (Trial 1) or reminded (Trials 2 and 3) of their own goal (“You are looking for the carrots that have orange inside”) and the demonstrator’s goal (Same Goals condition: “Rabbit is looking for carrots with orange inside”; Different Goals condition: “Bird is looking for carrots with worms inside”). To check participants’ understanding of their own goal and the demonstrator’s goal, they were asked four goal understanding questions (“What is inside the carrot that you are looking for?”; “What is inside the carrot that you are not looking for?”; “What is inside the carrot that Rabbit/Bird is looking for?”; “What is inside the carrot that Rabbit/Bird is not looking for?”). If there was no response, participants were prompted with the two possible answers by E1 (“orange” or “worms”). Due to a lack of verbal responses in 3-year-olds, it was necessary to offer an alternative to verbal responses; therefore, E1 held out examples of the capsule contents offering participants the option to point instead. Incorrect responses were not corrected. Participants were considered to have understood the task goals if they answered all four questions correctly in each trial.

Both participants and demonstrators were provided with three tokens. Before making a selection, both needed to pay a token. The purpose of the tokens was both to show whose turn it was and to make clear to children that they had multiple (but limited) attempts at the task, thereby highlighting the “costly” nature of each selection. The demonstrator always went first to provide the demonstration. Participants observed as the demonstrator paid a token and indicated a bucket from which E2 could select a capsule (only E2 was aware of the contents of each bucket prior to selection). Once a capsule had been selected, the demonstrator opened it slightly and peeked inside, ensuring that the contents were not visible to participants. The demonstrator then accepted or rejected the selected capsule; in the Same Goals condition Rabbit accepted carrots that were orange inside and rejected carrots that contained worms, and in the Different Goals condition Bird accepted carrots that contained worms and rejected carrots that were orange inside.

Following the demonstration, participants were encouraged to take their turn by paying a token and selecting a capsule from one of the buckets. Once they had selected a capsule they were instructed to peek inside and choose whether they wanted to keep it in their pan or not keep it and give it to E1 (participants chose to keep nontarget capsules in 11.6% of trials). To determine whether participants had correctly inferred the outcome of the demonstration, they were asked a demonstration outcome understanding question (“What was inside the carrot that Rabbit/Bird picked?”). At the end of each trial participants were also asked three memory questions, requiring them to recall the buckets from which they and the demonstrator selected capsules and the contents of their selected capsule.

### Statistical analysis

Analyses were performed using R (R Core Team, 2020), with generalized linear mixed effects analyses (GLMMs) being performed using the *lme4* package (Bates, Mächler, Bolker, & Walker, 2014) with logit regression. All *p* values < .05 were accepted as statistically significant. The binary dependent variables in the analyses were: goal understanding, demonstration outcome understanding, and use of an appropriate response. Where specified as fixed effects, the following variables were sum-coded: condition (Same Goals as −1, Different Goals as 1) and demonstration outcome (unsuccessful as −1, successful as 1). Age was centered and scaled to measure thousands of days. The random effects structure for each model aimed to include by-participant random slopes for all fixed effects and to keep random effects structures “maximal” where possible (following Barr, Levy, Scheepers, & Tily, 2013). Where the maximal model resulted in nonconvergent or singular fit models, random slopes were removed, followed by random intercepts where necessary, until a convergent nonsingular model was obtained. Post hoc analyses were carried out using estimated marginal means with the *emmeans* package (Lenth, Singmann, Love, Buerkner, & Herve, 2019). Post hoc results are given on the log odds ratio scale.

## Results

The key aim of this study was to determine whether children were able to take others’ goals into account when interpreting and using social information to achieve their own goal. We were particularly interested in whether children would find it more challenging to use information provided by a demonstrator whose goal was different from their own.

### Understanding

#### Goal understanding

**Fig. 2:**
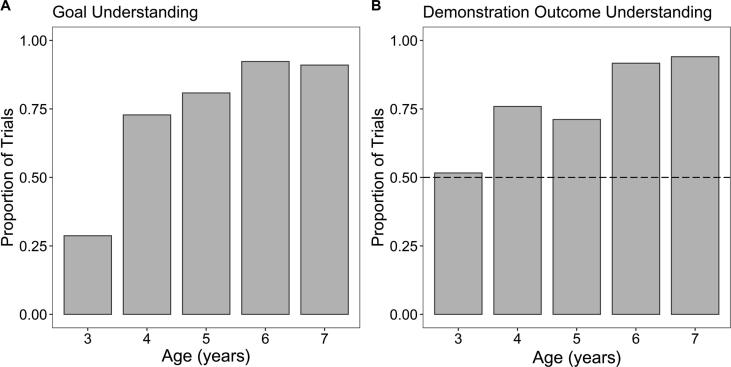
Proportions of trials in which children were considered to have each type of understanding in each age group. (A) Goal understanding (*N* = 570). Children were considered to have understood the task goals in a given trial if they answered all four goal understanding questions correctly. (B) Demonstration outcome understanding (including only the trials in which children demonstrated goal understanding; *N* = 420): Proportions of trials in which children correctly inferred the contents of the capsule that had been selected by the demonstrator. The dashed line indicates chance performance.

First, we looked at children’s understanding of their own goal and the demonstrator’s goal, which was expected to increase with age, we also expected that children’s understanding of the task goals would be better in the Same Goals condition. Children were considered to have understood the task goals in 74% of the 570 trials (answered all four goal understanding questions correctly within a trial). We compared children’s goal understanding with regard to the condition to which they were assigned (Same Goals or Different Goals) and age. A GLMM with a binary dependent variable for whether children passed the goal understanding questions in each trial was built with fixed effects of condition and age, the interaction between these variables, and a random intercept of participant ID. A significant main effect of age (*b* = 6.39, *SE* = 1.26, *z* = 5.09, *p* < .001) indicated that children’s goal understanding improved with age. A one-way analysis of variance (ANOVA) revealed significant differences in goal understanding between ages in years, *F*(4, 565) = 52.28, *p* < .001. Post hoc comparisons using Tukey HSD (honestly significant difference) tests indicated that 3-year-olds’ goal understanding was significantly poorer than that of all other ages (*p* < .001) and that 6- and 7-year-olds’ goal understanding was significantly greater than that of 4-year-olds (*p* ≤ .003). There was a particularly striking increase in goal understanding between 3-year-olds (*M* = .29, *SD* = .45, *n* = 108) and 4-year-olds (*M* = .73, *SD* = .45, *n* = 114) (Fig. 2A). The GLMM did not reveal evidence of an effect of condition (*b* = 0.28, *SE* = 0.45, *z* = 0.63, *p* = .529) or an interaction between condition and age (*b* = 1.20, *SE* = 1.02, *z* = 1.17, *p* = .241), suggesting that although goal understanding improved with age, there were no differences relative to the Same Goals and Different Goals manipulation.

We also looked at which of the goal understanding questions children who were not considered to have understood the task goals were getting wrong. Children failed to identify both their own goal and the demonstrator’s goal in 41.3% of the 150 trials. However, we found that children correctly identified their own goal but not the demonstrator’s goal in significantly more trials (37.3%) than they correctly identified the demonstrator’s goal but not their own goal (21.3%), *z* = 8.51, *p* = .002.

#### Demonstration outcome understanding

Looking only at the trials in which children demonstrated goal understanding (*n* = 420), we examined children’s understanding of the demonstration outcome (i.e., whether children correctly inferred the contents of the capsule that the demonstrator selected, as measured by their answer to the question “What was inside the capsule that Rabbit/Bird picked?”). Again, we expected understanding to increase with age and to be better in the Same Goals condition. Children correctly inferred the outcome of the demonstration in 81% of trials. A GLMM was built for demonstration outcome understanding with fixed effects of condition, demonstration outcome, age, the interactions among these variables, and a random intercept of participant ID. Results revealed that children’s understanding of the demonstration outcome improved significantly with age (*b* = 2.19, *SE* = 0.46, *z* = 4.72, *p* < .001) (Fig. 2B). Demonstration outcome understanding was significantly greater in trials with a successful demonstration (*M* = .87, *SD* = .34, *n* = 202; *b* = 0.41, *SE* = 0.17, *z* = 2.40, *p* = .016) compared with trials with an unsuccessful one (*M* = .76, *SD* = .43, *n* = 218). There was no evidence of an effect of condition (*b* = 0.02, *SE* = 0.19, *z* = 0.13, *p* = .896) or any interactions among age, condition, and demonstration outcome (*p* ≥ .213).

We analyzed children’s demonstration outcome understanding with regard to children’s age in years. A one-way ANOVA revealed significant differences in demonstration outcome understanding between ages, *F*(4, 415) = 12.40, *p* < .001. Post hoc comparisons using Tukey HSD tests indicated that 6- and 7-year-olds’ understanding was significantly greater than that of 3-, 4-, and 5-year-olds (*p* ≤ .03) and that 4-year-olds’ understanding was significantly greater than that of 3-year-olds (*p* = .016). Binomial tests revealed that the proportion of trials in which 4- to 7-year-olds correctly inferred the demonstration outcome was significantly greater than chance (*p* < .001), whereas 3-year-olds were no different from chance (*M* = .51, *SD* = .50, *n* = 31, *p* = 1.00).

### Appropriate information use

Appropriate information use was measured by the proportion of trials in which children selected a target capsule. It was predicted that appropriate information use would improve with age and that performance would be greater in the Same Goals condition than in the Different Goals condition. Overall, children responded appropriately in 60% of 570 trials. A GLMM was built for appropriate information use with fixed effects of condition, demonstration outcome, age, the interactions among these variables, random intercepts of goal understanding and demonstration outcome understanding, and a by-participant random slope for demonstration outcome. A significant main effect of age revealed that target capsule selections increased with age (*b* = 0.54, *SE* = 0.24, *z* = 2.26, *p* = .024). Although information use improved significantly with age, even 7-year-olds did not perform particularly well, selecting the target capsule in only 70% of trials. Binomial tests revealed that the older three ages selected the target capsule in significantly more trials than would be expected by chance: 5 years (*p* = .022), 6 years (*p* = .002), and 7 years (*p* < .001). By contrast, younger children were not different from chance (3 years: *p* = .387; 4 years: *p* = 1.00).

**Fig. 3:**
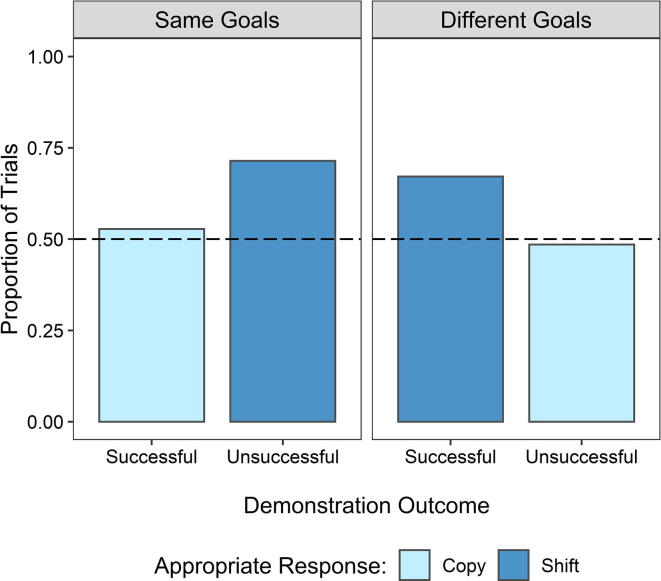
Proportions of trials in which children selected the target capsule by condition, demonstration outcome, and appropriate response. The dashed line indicates chance performance.

There was no evidence of an overall effect of condition (*b* = −0.12, *SE* = 0.10, *z* = −1.14, *p* = .253) or demonstration outcome (*b* = −0.02, *SE* = 0.11, *z* = −0.22, *p* = .827) on appropriate information use. However, a significant two-way interaction between condition and demonstration outcome (*b* = 0.47, *SE* = 0.11, *z* = 4.44, *p* < .001) indicated that appropriate information use was dependent on both the condition and the demonstration outcome. To clarify this interaction, we performed a post hoc analysis using *emmeans.* This revealed that appropriate information use was significantly greater in the Same Goals condition following unsuccessful demonstrations (*b* = 0.99, *SE* = 0.31, *z* = 3.21, *p* = .001) and in the Different Goals condition following successful demonstrations (*b* = 0.89, *SE* = 0.29, *z* = 3.04, *p* = .002). This pattern relates to the appropriate use of two possible response types: copying and shifting. Children appropriately used the copy response in only 51% of 280 trials. By contrast, children appropriately used the shift response in 69% of 290 trials (Fig. 3). Shifting was the appropriate response in the Same Goals condition following unsuccessful demonstrations (71% appropriate responses in 147 trials) and in the Different Goals condition following successful demonstrations (67% appropriate responses in 143 trials).

**Fig. 4:**
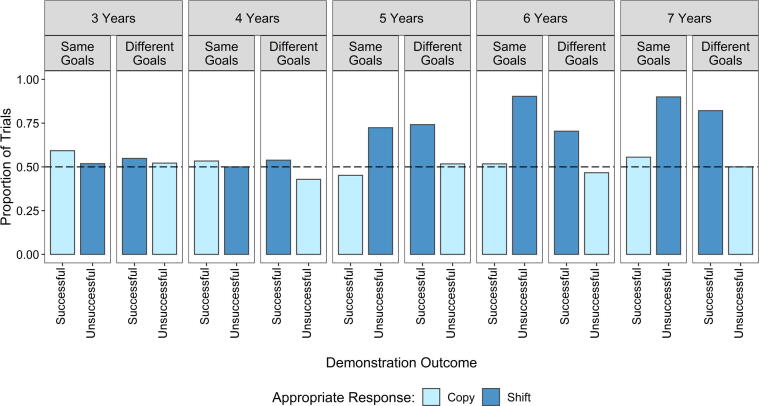
Proportions of trials in which children selected the target capsule by age in years, condition, demonstration outcome, and appropriate response. The pattern of results observed in Fig. 3 was evident only in 5-, 6-, and 7-year-old children, whereas 3- and 4-year-old children’s use of both types of appropriate response was no different from chance. The dashed line indicates chance performance.

The model also revealed a significant three-way interaction among condition, demonstration outcome, and age (*b* = 0.76, *SE* = 0.21, *z* = 3.64, *p* < .001). Post hoc analysis using *emmeans* showed that this interaction highlights the difference between the two response types with regard to age; appropriate copying did not improve with age, remaining close to chance, whereas appropriate shifting increased with age (see Fig. S1 and post hoc results in the supplementary material). Separating information use by age in years relative to the appropriate response required in each condition following each demonstration type (Fig. 4) showed that 3- and 4-year-olds did not follow the same pattern of appropriate responses as 5-, 6-, and 7-year-olds. It is worth highlighting that 3- and 4-year-olds’ information use was no different from chance following both conditions and demonstration types, whereas 5-, 6-, and 7-year-olds’ information use was greater than chance only when the appropriate response was to shift. This pattern of results suggests that there may be an age-related change in the way that children approach the use of social information; in this sample this change appears to occur between 4 and 5 years of age.

For completeness, we repeated our analysis of appropriate information use including only the trials in which children had demonstrated goal understanding and demonstration outcome understanding (*n* = 342). A GLMM was built for appropriate information use with fixed effects of condition, demonstration outcome, age, the interactions among these variables, and a by-participant random slope for demonstration outcome. In contrast to the previous model, there was no evidence of an overall effect of age (*b* = 0.42, *SE* = 0.31, *z* = 1.36, *p* = .175). Binomial tests revealed that the older four ages selected the target capsule in significantly more trials than would be expected by chance (4 years: *p* = .023; 5 years: *p* = .029; 6 and 7 years: *p* < .001). By contrast, 3-year-olds were not different from chance (*p* = .210). As in the full sample, the model revealed a significant two-way interaction between condition and demonstration outcome (*b* = 0.43, *SE* = 0.15, *z* = 2.94, *p* = .003) and a significant three-way interaction among condition, demonstration outcome, and age (*b* = 0.85, *SE* = 0.33, *z* = 2.57, *p* = .010). Post hoc analyses using *emmeans* indicated that appropriate information use was greater when the appropriate response was to shift and improved with age only when the appropriate response was to shift in the Same Goals condition (post hoc results are given in the supplementary material).

### Memory check

Children recalled the location of their own capsule selection in 93% of the 570 trials, with similar rates across all ages (84%–97%). Children recalled the location of the demonstrator’s selection in 85% of trials and showed age-related differences in recall: the older ages (5-, 6-, and 7-year-olds) successfully recalled in ≥ 90% of trials, and the younger ages (3- and 4-year-olds) successfully recalled in 69% and 78% of trials, respectively. A one-way ANOVA revealed significant differences in recall between ages, *F*(4, 565) = 11.11, *p* < .001. Post hoc comparisons using Tukey HSD tests indicated that 5-, 6-, and 7-year-olds’ recall was significantly greater than that of 3- and 4-year-olds (*p* ≤ .045).

Removing the trials in which children failed to recall the location of the demonstrator’s selection from the appropriate information use model produced the same pattern of results as looking only at the trials in which children demonstrated goal understanding and demonstration outcome understanding (results are given in the supplementary material).

### Explicit verbal reasoning

We examined children’s responses to the explicit verbal reasoning question regarding how they were choosing from which bucket to select a capsule. Each child gave a single response after all three trials had been completed. Responses were categorized into three levels: (1) nonreasoned responses (including incorrect responses, incoherent responses, and no responses), (2) partially reasoned responses (where explanations included some evidence of explicit task understanding but were incomplete), and (3) fully reasoned responses (where explanations provided clear evidence of explicit task understanding).

Of the 190 children, only 7 were categorized as having provided fully reasoned responses. Older children gave more reasoned responses than younger children (Table 1). The poor rate of explicit verbal reasoning suggests that children found articulating justifications for their choices to be particularly challenging despite older children displaying high levels of goal understanding and demonstration outcome understanding.

**Table 1:** Number of children providing each category of response to the explicit verbal reasoning question by age in years.

		Age (years)				
Response category	Total	3	4	5	6	7
Nonreasoned	172	36	37	34	35	30
Partially reasoned	11	–	1	5	2	3
Fully reasoned	7	–	–	1	2	4

### Adult participants

As even the oldest children used the appropriate response in only 70% of trials and age-related improvements were restricted to trials in which the appropriate response was to shift, it was important to examine our assumption that adults would employ appropriate responses to achieve a goal. Therefore, we conducted an exploratory follow-up study using the same methods with adults (6 in each condition; *n* = 12). Adults used the appropriate response to select the target capsule in 33 of the 36 trials (92%), and all gave fully reasoned responses to the explicit verbal reasoning question. Thus, consistent with our expectations, adults responded appropriately regardless of the response required. Adults were recruited at the University of Stirling and received £3 compensation for their participation.

## Discussion

The methods employed within this study allowed us to examine the developmental trajectory of children’s capacity to understand others’ goals relative to their own, their ability to use this knowledge to infer the outcomes of social demonstrations, and their ability to apply this understanding to strategically adopt an appropriate response.

Contrary to our predictions, we discovered that there was no effect of the Same Goals and Different Goals manipulation on children’s understanding of the task goals, children’s ability to infer the outcome of the demonstration, or children’s appropriate use of social information. That is, whether participants’ goals and the demonstrator’s goals were aligned or not did not predict children’s performance in any aspect of the task, suggesting that children were not specifically impacted by the need to account for others’ conflicting goals.

With regard to goal understanding, we found that the proportion of trials in which children were considered to have understood both their own task goals and the demonstrator’s task goals increased with age. A particularly large improvement in goal understanding between 3-year-olds and 4-year-olds indicated a developmental jump in children’s capacity to recognize, represent, and report others’ goals. Although evidence regarding the age at which children develop an understanding of others’ diverse desires is inconsistent in the literature, the lack of difference between the conditions in the current study suggests that from 4 years of age children were capable of understanding the demonstrators’ goals even when they conflicted with their own. We also discovered that children who did not demonstrate goal understanding found identifying others’ goals to be more challenging than identifying their own goals.

As the outcome of the demonstration was opaque to the participants, we examined their ability to interpret it using their understanding of the demonstrator’s goal and the demonstrator’s reaction to the capsule. To infer the demonstrator’s reaction to the selected capsule, children needed to recognize that the demonstrator had perceptual access via peeking and therefore had privileged knowledge about the contents of the capsule to make an informed choice. Children’s capacity to interpret the content of social information was found to improve with age. Older children were found to correctly infer the outcome of the demonstration in a greater proportion of trials than younger children. Whereas 4- to 7-year-olds correctly inferred the outcome of the demonstrations significantly more often than would have been expected by chance, only from 6 to 7 years were they able to *reliably* infer the correct outcome.

We suggest that there are two possible explanations for the age-related improvement in the correct interpretation of the demonstration outcome. It could be related to the capacity to account for others’ goals, or it may be related to understanding of access to information and knowledge formation. Literature suggests that children do not demonstrate understanding of how knowledge is formed with consideration of the role of access to information until around 6 years of age (Kloo & Rohwer, 2012). Therefore, older children’s more reliable interpretations could be linked to the ability to reason about others’ access to information. That children correctly inferred the demonstration outcome following successful demonstrations suggests that some children may have used less cognitively demanding processes such as biases based on more salient cues (in this case children may have used a positive cue from the demonstrator’s choice to keep the capsule). This could give the appearance of understanding that someone is knowledgeable due to the person’s access to information when in reality information access has not been considered. This may go some way toward explaining why younger children appeared to have understood the outcome of the demonstration but were not able to use the information.

The analysis of the full sample showed evidence of the expected age-related improvement in children’s appropriate social information use. At first glance this age-related improvement in appropriate information use could be seen as evidence that older children had recognized the value of the information that had been provided by others and were capable of using their understanding of the task goals and their interpretation of the outcome of the demonstration. However, even the oldest children’s performance was not particularly high, suggesting that the task paradigm was more challenging than anticipated. Indeed, when the trials in which children did not demonstrate task goal understanding or interpreted the outcome of the demonstration incorrectly were removed prior to analysis, there was no longer evidence of an age-related improvement in appropriate information use. The lack of an age effect in the reduced sample highlights that not having the capacity to understand others’ goals or to interpret the content of social information likely impedes on younger children’s ability to make use of available social information.

Surprisingly, the results of both models revealed a distinct difference in appropriate social information use related to the appropriate response type (i.e., copying and shifting). We found that appropriate social information use was restricted to when the appropriate response was to shift, with appropriate copying responses remaining at chance level. This pattern of results was also related to children’s age. Specifically, 3- and 4-year-olds’ appropriate information use did not follow the same pattern as that of the older children. While 3- and 4-year-olds’ performance remained no different from chance for both response types, 5-, 6-, and 7-year-olds’ performance was no different from chance for copying responses but was significantly above chance for shifting responses. Therefore, the age-related improvement in children’s use of appropriate responses was restricted to trials in which the appropriate response was to shift. The chance performance of younger children may have been in part due to high cognitive demands taxing their executive functions such as memory. Indeed, we found that they recalled from which location the demonstrator had selected a capsule less often than the older children. However, when only the trials in which children correctly recalled the location from which the demonstrator had selected a capsule were included in the analysis, the pattern of results remained the same.

Although the results suggest that children can account for others’ conflicting motivations, the restriction of appropriate information use to shift responses suggests that some children had not recognized the relevance of the social information for achieving their own goals. Rather, they may have been relying on implicit biases. The adult sample offers a useful comparison for what to expect from someone who has recognized the value of the information available in the demonstration as we are confident adults can do. Therefore, the distinct difference between the performance of the oldest children and the adults supports our interpretation that taking others’ goals into account in appropriate social information use is cognitively challenging whether they are aligned with one’s own goals or not. The poor rate of explicit verbal reasoning is consistent with this interpretation.

Alternatively, this pattern of results may be an artifact of the binary choice task. Other tasks that have used a logically similar binary choice reward structure have also reported better performance in children when the appropriate response was to shift, albeit in slightly younger populations than the sample tested here (e.g., Atkinson et al., 2021). In a binary choice task, where the demonstrator and participant share the same goal, a shift response following an unsuccessful demonstration performs an explore function that does not need to be traded off against an exploit option because the participant already knows that the alternative is undesirable. By contrast, appropriate copying following a successful demonstration involves a trade-off between explore and exploit motivations. When the demonstrator and participant have different goals, the opposite applies. Overall, this will inevitably favor shift responses (Gopnik, 2020). Evidence does suggest that children often choose to shift even in situations where they might benefit from copying (Blanco & Sloutsky, 2021). Thus, it is possible that even in cases where children recognize the value of the social information and can identify the appropriate response to achieve their goal, the motivation to shift may override the motivation to achieve the goal, making it difficult to ascertain the reason for particular choices.

Previous research into social information use has focused on copying responses. However, experimental paradigms in which the demonstrated solution is always successful or the successful response is to match one of the demonstrated solutions (e.g., Carr et al., 2015, van Leeuwen et al., 2018) make it challenging to distinguish between preferences for shifting and a failure to copy. Therefore, researchers previously may have concluded that children have failed to use the available social information to inform their responses when an alternative solution has been employed. Recent evidence highlights that in these cases children might be driven by a preference for shifting to alternative options rather than simply copying the options presented to them (e.g., Atkinson et al., 2021). Children who do not copy may in fact be fully cognizant of the social (or asocial; Blanco & Sloutsky, 2021) information available (i.e., potentially perfectly capable of producing the demonstrated response) but applying this with a motivation to shift. Indeed, in the current study the inclusion of both successful and unsuccessful demonstrations highlighted a distinct difference in children’s performance depending on whether the appropriate response was to copy or shift. Older children performed better at our task, but if we had included only successful demonstrations involving aligned goals, this would not have been apparent.

We suggest that this motivation to shift should also be distinguished from favoring information acquired through personal experience over that acquired from a social source, which has been a common interpretation of social learning studies that found low rates of copying. In the context of a social learning task, shift responses could reflect a preference for using personally acquired information over socially acquired information. However, in the absence of comparison with an individual learning condition, it is impossible to distinguish between a preference for personal (over social) information from a preference for exploring new (over known) options. Indeed, recent research shows that children’s preference for shifting is not peculiar to contexts of social information use; it was applied just as strongly following personally acquired and social information (Atkinson et al., 2021, Renner et al., 2021). Similarly, Blanco and Sloutsky (2021) found a preference for exploration in an individual learning task even when children might benefit from exploitation. Such findings suggest that shift responses could indicate that children are motivated by a preference for new information. The results of the current study are consistent with this interpretation.

We cannot rule out that the lower performance of younger children in this study could be attributed, at least in part, to high task demands in addition to those related to memory outlined above. For instance, participants’ assigned and natural goals may have been in conflict, posing a potential challenge for younger children whose inhibitory control is less well developed (Carlson and Moses, 2001, Gerstadt et al., 1994). Although they were told that all the capsules were the same in each bucket, it is possible that some children might not have believed or understood this explanation or might have forgotten that this was the case, thereby potentially affecting their ability to infer the demonstration outcome. Undoubtedly there is likely to be a developmental progression in children’s ability to reason about what the outcome of others’ choices reveals about the environment more generally. However, this may be a realistic challenge of social information use in many contexts and, as such, may be one of the factors that renders appropriate use of social information nontrivial even in situations where goal understanding is established, demonstration outcomes are inferred correctly, and memory is accurate.

The findings reported in the current study suggest that children are capable of understanding and representing others’ goals from around 4 years of age whether others’ goals are aligned with their own goals or not. We also demonstrated that children younger than 6 years find it difficult to infer, from others’ reactions, the content of social information when the outcome or reward value is opaque. Older children’s more consistent performance was likely underpinned by reasoning-based understanding of the demonstrators’ access to information via peeking. Younger children’s lower understanding did not appear to be related to understanding others’ goals. Rather, better performance following successful demonstrations indicated that some children were perhaps relying on less cognitively demanding biases based on more salient cues such as success.

To answer our original question, the findings suggest that learning from someone whose goal is not aligned with one’s own goal is no more cognitively challenging than learning from someone who has the same goal. Rather, it appears that taking another’s goals into account in appropriate social information use at all is cognitively challenging. Even at 7 years of age, children did not demonstrate an adult-like ability to account for another’s goal in social information use. Thus, even if children have the ability to interpret the content of social information, they may still struggle to recognize its relevance for achieving their own goal. In the absence of this recognition children’s choices may have instead been determined by heuristic biases and motivations toward exploration. Indeed, our most striking discovery was that appropriate social information use was restricted to cases in which the appropriate response was to shift, or to *avoid copying,* the demonstrator’s selection. This finding is seemingly in contrast to the vast majority of the social learning literature.

The late development and significant cognitive challenge associated with the ability to use social information about others’ goals suggests that human adults’ social information use is something quite special. That is, the flexibility afforded by the ability to recognize its value may offer a significant advantage in social information use that distinguishes human social learning from that of other animals. This kind of social information use therefore may be implicated in distinctively human cumulative culture.

### CRediT authorship contribution statement

**Kirsten H. Blakey:** Conceptualization, Methodology, Formal analysis, Investigation, Project administration, Writing – original draft, Writing – review & editing. **Mark Atkinson:** Formal analysis, Writing – review & editing. **Eva Rafetseder:** Conceptualization, Writing – review & editing. **Elizabeth Renner:** Formal analysis, Writing – review & editing. **Christine A. Caldwell:** Conceptualization, Methodology, Writing – review & editing, Funding acquisition, Supervision.

## Declaration of Competing Interest

The authors declare that they have no known competing financial interests or personal relationships that could have appeared to influence the work reported in this paper.
