# Supplementary material for: Taking account of others’ goals in social information use: Developmental changes in 3- to 7-year-old children
Source: J Exp Child Psychol. 2022 Mar;215:105325. doi: 10.1016/j.jecp.2021.105325 (PMC8784822; doi:10.1016/j.jecp.2021.105325)
Supplement: Supplementary data 1 [file mmc1.docx]

# Taking account of others’ goals in social information use: Developmental changes in 3- to 7-year-old children – Supplementary information

## Results

###
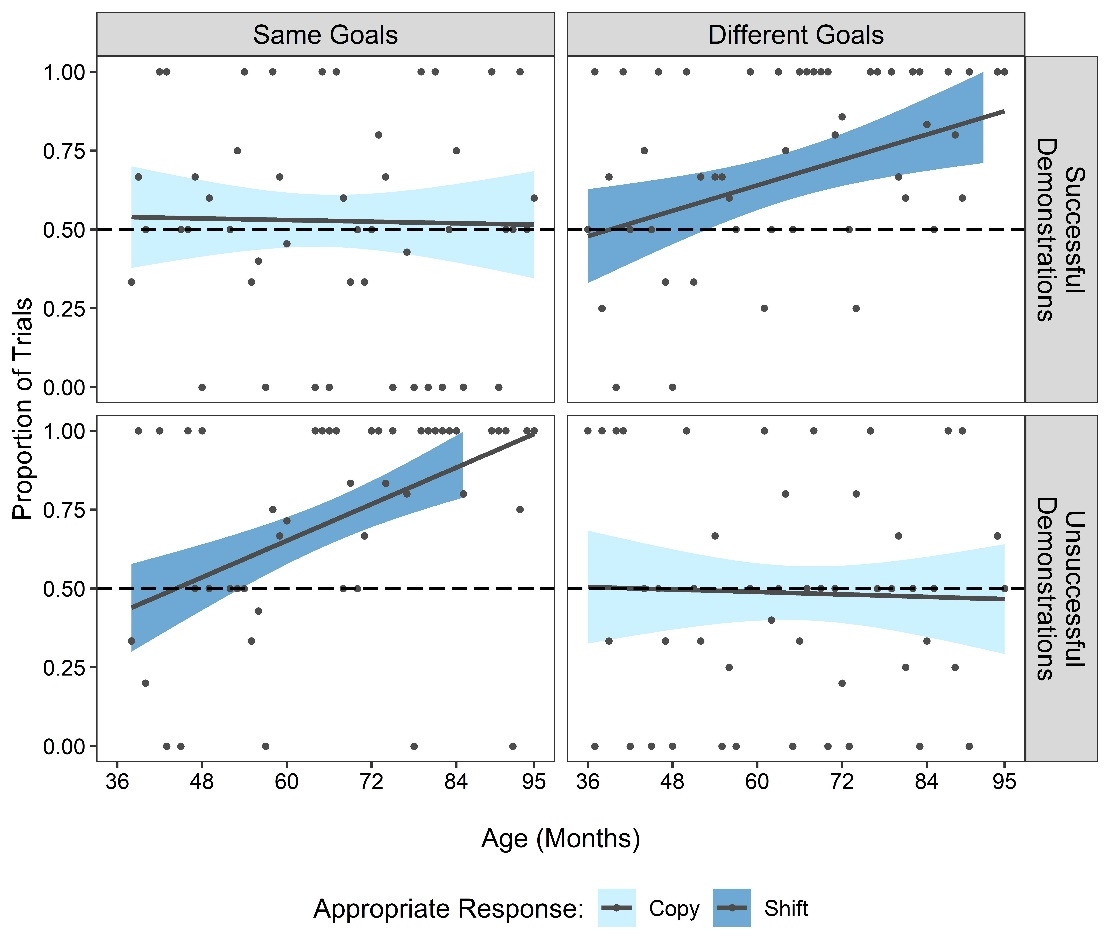
Appropriate information use

Note. Dashed line indicates chance performance.

**Figure S1**

*Proportion of Trials in Which Children Selected the Target Capsule by Condition, Demonstration Outcome, Age in Months, and Appropriate Response.*

In the main manuscript, we reported the results of a GLMM for appropriate information use. To clarify a three-way interaction between condition, demonstration outcome, and age, we carried out a post hoc analysis using *emmeans*. The three-way interaction indicates a difference in use of appropriate response types (copying and shifting) related to age. Appropriate information use was only greater in older children when the appropriate response was to shift, while there was not an age-related difference for appropriate copying. As outlined in the main manuscript, the appropriate response was dependent on the condition and the demonstration outcome. The results are shown in Supplementary Table S1. Results are given on the log odds ratio scale.

**Table S1**

*Pairwise Contrasts for the Interaction Between Condition, Demonstration Outcome, and Age in the Appropriate Information Use Analysis*

| Condition | Demonstration Outcome | Age Quartiles | Estimate | SE | *Z* ratio | *p* value |
| --- | --- | --- | --- | --- | --- | --- |
| Full sample (*N* = 570) | | | | | | |
| Same Goals | Successful | Upper – lower | −0.04 | 0.08 | −0.53 | .595 |
|  | Unsuccessful | Upper – lower | 0.27 | 0.08 | 3.66 | < .001* |
| Different Goals | Successful | Upper – lower | 0.17 | 0.08 | 2.30 | .022* |
|  | Unsuccessful | Upper – lower | −0.05 | 0.09 | −0.58 | .561 |
| Passed understanding questions sample (*N* = 342) | | | | | | |
| Same Goals | Successful | Upper – lower | −0.09 | 0.10 | −0.89 | .372 |
|  | Unsuccessful | Upper – lower | 0.15 | 0.07 | 2.16 | .031* |
| Different Goals | Successful | Upper – lower | 0.13 | 0.08 | 1.63 | .104 |
|  | Unsuccessful | Upper – lower | −0.06 | 0.11 | −0.57 | .568 |

### Memory check

Given the age differences in children’s recall of the location of the demonstrator’s selection we repeated the information use analysis including only the trials in which children’s recall was correct (*n* = 496). A GLMM was built for appropriate information use with fixed effects of condition, demonstration outcome, age, the interactions between these variables, and random intercepts of goal understanding, demonstration outcome understanding, and participant ID. In line with the previous analysis, there was a significant two-way interaction between condition and demonstration outcome (*b =* 0.51, *SE* = 0.10, *z* = 4.88, *p* < .001) and a significant three-way interaction between condition, demonstration outcome and age (*b =* 0.85, *SE* = 0.22, *z* = 3.90, *p* < .001). Post hoc analysis using *emmeans* indicated that information use was greater and only improved with age when the appropriate response was to shift. The results are shown in Supplementary Table S2. Results are given on the log odds ratio scale. However, the main effect of age did not reach significance (*b =* 0.45, *SE* = 0.25, *z* = 1.78, *p* = .0748), which suggests that demands on memory in younger children may account for at least some of the age effect in the original appropriate information use model.

| Condition | Demonstration Outcome | Age Quartiles | Estimate | SE | *Z* ratio | *p* value |
| --- | --- | --- | --- | --- | --- | --- |
| Same Goals | Successful | Upper – lower | −0.01 | 0.08 | −1.25 | .211 |
|  | Unsuccessful | Upper – lower | 0.26 | 0.08 | 3.39 | < .001* |
| Different Goals | Successful | Upper – lower | 0.15 | 0.07 | 2.11 | .035* |
|  | Unsuccessful | Upper – lower | −0.06 | 0.08 | −0.73 | .465 |

**Table S2**

*Pairwise Contrasts for the Interaction Between Condition, Demonstration Outcome, and Age in the Appropriate Information Use Analysis (Passed Memory Question Sample)*

## Verbal script

### Same Goals Condition

1. “You are going to play a game with Rabbit.”

E1 presents 1^st^ set of buckets with carrots inside.

1. “Look, we have 2 buckets of carrots, in one of the buckets the carrots are orange inside, like this one (E1 shows carrot with orange inside), and in the other bucket the carrots have worms inside, like this one (E1 shows carrot with worm inside).”
2. “You are looking for the carrots that have orange inside to put in your pan (E1 gives pan). Rabbit likes to eat carrots that are orange inside, so Rabbit is looking for carrots with orange inside to put in the basket (E1 presents basket).”
3. “What is inside the carrots that you are looking for?”
   “What is inside the carrots that you are **not** looking for?”

“What is inside the carrots that Rabbit is looking for?”
“What is inside the carrots that Rabbit is **not** looking for?”

1. “You and Rabbit both have 3 tokens; you need to pay a token into the box before you can pick a carrot from one of the buckets.”
2. “It is Rabbit’s turn first, Rabbit is going to pay a token into the box, and Rabbit is going to choose a bucket to pick a carrot from (Rabbit indicates bucket for E2 to select a carrot from). Rabbit is going to have a peek inside the carrot and decide to keep it in the basket, or not keep it and give it to me.” Carrot opened by E2 so Rabbit can peek inside without the child seeing the contents.
3. “Now it is your turn.”

E1 guides child through their turn.

1. “You can pay a token into the box, and you can pick a carrot from one of the buckets, now you can have a peek inside and decide if you want to keep it and put it in your pan, or not keep it and give it to me.”
   Child can accept or reject either kind of carrot, E1 provides no feedback.
2. “What was inside the carrot that Rabbit picked?”
3. “Which bucket did they look in?”
4. “What was inside the carrot that you picked?”
5. “Which bucket did you look in?”

Child keeps any carrots they find in their pan; Rabbit’s carrots stay in basket.

E1 takes buckets out of sight, refills them, changes lid colors, and randomly swaps sides.

1. “Look you have 2 tokens left; would you like to play the game again?”

Trial 2 follows same pattern as trial 1 (from step 3). Trial 3 follows trial 2 in same pattern. Game finishes after 3 trials.

1. “How were you deciding which buckets to choose?”

### Different Goals Condition

1. “You are going to play a game with Bird.”

E1 presents 1^st^ set of buckets with carrots inside.

1. “Look, we have 2 buckets of carrots, in one of the buckets the carrots are orange inside, like this one (E1 shows carrot with orange inside), and in the other bucket the carrots have worms inside, like this one (E1 shows carrot with worm inside).”
2. “You are looking for the carrots that have orange inside to put in your pan (E1 gives pan). Bird likes to eat worms, so Bird is looking for carrots with worms inside to put in the basket (E1 presents basket).”
3. “What is inside the carrots that you are looking for?”
   ”What is inside the carrots that you are **not** looking for?”

“What is inside the carrots that Bird is looking for?”
“What is inside the carrots that Bird is **not** looking for?”

1. “You and Bird both have 3 tokens; you need to pay a token into the box before you can pick a carrot from one of the buckets.”
2. “It is Bird’s turn first, Bird is going to pay a token into the box, and Bird is going to choose a bucket to pick a carrot from (Bird indicates bucket for E2 to select a carrot from). Bird is going to have a peek inside the carrot and decide to keep it in the basket, or not keep it and give it to me.” Carrot opened by E2 so Bird can peek inside without the child seeing the contents.
3. “Now it is your turn.”
   E1 guides child through their turn.
4. “You can pay a token into the box, and you can pick a carrot from one of the buckets, now you can have a peek inside and decide if you want to keep it and put it in your pan, or not keep it and give it to me.”
   Child can accept or reject either kind of carrot, E1 provides no feedback.
5. “What was inside the carrot that Bird picked?”
6. “Which bucket did they look in?”
7. “What was inside the carrot that you picked?”
8. “Which bucket did you look in?”

Child keeps any carrots they find in their pan; Bird’s carrots stay in basket.

E1 takes buckets out of sight, refills them, changes lid colors, and randomly swaps sides.

1. “Look you have 2 tokens left; would you like to play the game again?”

Trial 2 follows same pattern as trial 1 (from step 3). Trial 3 follows trial 2 in same pattern. Game finishes after 3 trials.

1. “How were you deciding which buckets to choose?”
